# Factors Associated with Higher Sitting Time in General, Chronic Disease, and Psychologically-Distressed, Adult Populations: Findings from the 45 & Up Study

**DOI:** 10.1371/journal.pone.0127689

**Published:** 2015-06-03

**Authors:** Ronald C. Plotnikoff, Sarah A. Costigan, Camille Short, Anne Grunseit, Erica James, Natalie Johnson, Adrian Bauman, Catherine D’Este, Hidde P. van der Ploeg, Ryan E. Rhodes

**Affiliations:** 1 Priority Research Centre for Physical Activity and Nutrition, The University of Newcastle, Newcastle, New South Wales, Australia; 2 Centre for Physical Activity Studies (CPAS), Institute for Health and Social Science Research, Central Queensland University, Rockhampton, Queensland, Australia; 3 Prevention Research Collaboration, School of Public Health, The University of Sydney, Sydney, New South Wales, Australia; 4 School of Medicine & Public Health Discipline of Health Behaviour Science, Faculty of Health and Medicine, The University of Newcastle, Newcastle, New South Wales, Australia; 5 Research School of Population Health ANU College of Medicine, National Centre for Epidemiology & Population Health, The Australian National University, Canberra, Australian Capital Territory, Australia; 6 Department of Public and Occupational Health, EMGO Institute for Health and Care Research, VU University Medical Centre, Amsterdam, The Netherlands; 7 Behavioural Medicine Laboratory, School of Exercise Science, University of Victoria, Victoria, British Columbia, Canada; UKK Institute for Health Promotion, FINLAND

## Abstract

This study examined factors associated with higher sitting time in general, chronic disease, and psychologically-distressed, adult populations (aged ≥45 years). A series of logistic regression models examined potential socio-demographic and health factors associated with higher sitting (≥6hrs/day) in adults from the 45 and Up Study (n = 227,187), including four separate subsamples for analysis comprising those who had ever had heart disease (n = 26,599), cancer (n = 36,381), diabetes (n = 19,550) or psychological distress (n = 48,334). Odds of higher sitting were significantly (p<.01) associated with a number of factors across these groups, with an effect size of ORs≥1.5 observed for the high-income ≥$70,000AUD, employed full-time and severe physical limitations demographics. Identification of key factors associated with higher sitting time in this population-based sample will assist development of broad-based, public health and targeted strategies to reduce sitting-time. In particular, those categorized as being high-income earners, full-time workers, as well as those with severe physical limitations need to be of priority, as higher sitting appears to be substantial across these groups.

## Introduction

Current evidence clearly demonstrates the health benefits of adults participating in at least 30 minutes of moderate-vigorous physical activity (MVPA) on most days of the week [1]. However, recent research has shown that even when adults meet physical activity recommendations, there may be adverse metabolic and health effects from prolonged sitting [2]. Sedentary behavior and physical activity are measured using their metabolic equivalent (MET), with one MET characterising the energy expended when sitting quietly [1]. Prolonged sedentary behavior (sitting or reclining, 1–1.5METS) [3] independently affects health and wellbeing [4–8] due to reduced energy expenditure and a lack of muscular activity [9], and has been linked to all-cause mortality and possibly overweight/obesity, cardiovascular disease, adverse metabolic profiles, osteoporosis, type two diabetes, insulin resistance and various cancers [6, 10, 11] as well as reduced psychological and social functioning [12].

In Australia, population-based self-report data indicates that adults spend approximately four hours/day engaging in sedentary leisure activities, with 30% spending more than five hours/day [13–15]. However, objective assessment (i.e., accelerometer data) suggests that adults on average spend more than half their waking hours in sedentary activities (primarily prolonged sitting, including work related activities) [16, 17].

Due to the health risks and high prevalence of sedentary behavior, higher sitting time has been identified as a global public health concern [3]. Examination of factors associated with prolonged sitting is needed to inform cost-effective and sustainable primary and secondary prevention interventions. While some research has been conducted [10, 15, 18–21], additional exploration of populations with specific chronic disease or psychological distress [22] is needed to determine whether population-based interventions should be tailored to major disease groups versus generic ‘one-size-fits-all’ approaches [5]. The objective of this study is to examine factors associated with higher sitting time behavior in (i) general, (ii) chronic disease (heart disease/cancer/diabetes), and (iii) psychologically distressed adult populations, among a large sample of Australian adults.

## Methods

The 45 and Up Study received ethics approval from the University of NSW Human Research Ethics Committee. The University of Newcastle Human Research Ethics Committee approved this secondary analysis (H-2014-0042).

### Study population

The study used baseline data from The 45 and Up Study [23], a large-scale prospective cohort study (data collected January 2006-December 2009) of over 260,000 individuals aged 45 years or older living in New South Wales, Australia, with data on socio-demographic characteristics, health conditions, and lifestyle behaviors [24], collection via a pen and paper self-report questionnaire.

### Sitting Time

Sitting time was determined using a single item “About how many hours in each 24 hour day do you usually spend sitting?”. This measure is equivalent to the International Physical Activity Questionnaire (IPAQ) sitting time assessment [25]. A number of population-based studies have employed a single-item assessment of total sitting time[26]; such studies have consistently reported associations between high sitting time and poor health/mortality[27, 28]. Participants responses were then dichotomized as ‘low sitting time’ (≤5.5 hours/day) versus ‘higher sitting time’ (>5.5 hours/day) based on the sample’s mean and related studies [24, 29]. Effectively resulted in a dichotomy of <6 vs ≥6 hours for low and higher sitting time respectively. A recent study examining the United States National Health and Nutrition Examination Survey (NHANES) data also employed a similar cut-point (i.e., 6 hours) to examine the association between sitting time and cardiometabolic risk [29]. Further, a very recent study also employed a 6-hour cut-point to examine the excess sitting time and risk of heart disease and all-cause mortality [30]. Moreover, George and colleagues [24] reported an increased risk of chronic diseases of >4 hours of sitting per day from study participants, with greater risks of 6 or more hours of sitting time.

### Demographic variables

Demographic variables of interest included: age (derived from self-reported date of birth and categorized as 45–54, 55–64, 65–74 and 75+ years); education (no school certificate or other qualification and school or intermediate certificate, 12 years of schooling and/or non-degree certification, and university degree); marital status (married/defacto/partnered or single/widowed/divorced); annual household income (≤$10,000, $10,000–$29,999, $30,000–$69,999, >$70,000 AUD) (note: the average full-time Australian income is $74,724 per annum, before tax (ABS)) [19]; work status (not working, working part-time, working full-time) and postcode (derived from the respondents' postcode and classified according to the Australian Standard Geographical Classification (ASGC) Remoteness Index) [31].

### Clinical Variables

Body mass index (BMI) was calculated from self-reported weight and height, which has shown excellent agreement with objectively measured BMI categories [32], and categorized as: underweight (BMI:≤18.5); healthy weight (BMI:18.5–24.9); overweight (BMI:25.0–29.9); and, obese (BMI:⩾30). Co-morbidities were assessed by participants reporting any previous diagnosis of heart disease, diabetes mellitus, and various cancers (other than non-melanoma skin cancers) by a physician. Self-rated health was assessed with a single question on a 5-point scale (ranging from poor-excellent) from the 36-item Short Form Health Survey [33]. Health limitations (function) were assessed using the Medical Outcomes Study Physical Functioning scale, which assesses the extent to which an individual’s health limits their ability to perform daily functional activities and classified as: no limitation (100); minor limitation (95–99); mild limitation (85–94); moderate limitation (60–84); and severe limitation (0–59) [34].

### Health Behaviors

Physical activity was assessed with the Active Australia Survey [35] which measures minutes of walking and other moderate and vigorous physical activity in the past week, and has acceptable reliability and validity [36, 37]. Individuals were categorized as follows: no physical activity (0 min/week), some physical activity but not meeting recommended levels (1–149 min/week), meeting the minimum but less than twice the amount of the WHO recommendation (150–299 min/week) [38] and meeting high levels of activity (>300 min/week) [39]. Smoking status (current smoker/former smoker/never smoked) was classified based on responses to the following single item questions (with yes/no response options): ‘Have you ever been a regular smoker’ and ‘Are you a regular smoker now?’ [40]. Alcohol consumption was obtained using a single open-ended response item asking participants ‘About how many alcoholic drinks do you have each week?’ and was classified as no drinks; 1–7 drinks; 8–14 drinks; or ≥15 drinks per week [40].

### Psychosocial Measures

Prevalence of psychological distress was assessed using the Kessler Psychological Distress Scale (K10) [41, 42] incorporating ten questions regarding the respondent’s psychological state over the past four weeks. Total scores ranging from 10 to 50, were classified as: no/low emotional disturbances (<16); moderate emotional disturbances (16–21); and high/very high emotional disturbances (22+), consistent with previous research [43].

### Analysis

#### Missing data

Where the proportion of missing data for any covariate exceeded 5% (BMI, physical functioning, income and K10), a separate “missing” category was included in the analyses [28]. There were 18,671 (7%) missing on one or more of the remaining covariates, and 20,968 (7.9%) with data missing for sitting (either alone n = 11790 or also on a covariate). There was little difference in the rate of missing data on the covariates between the two sitting categories (low: 8.3%; high: 6.7%). Higher (>5%) than average rates of data missing for sitting were observed only among those aged 75 years or older (14.8% missing sitting) and/or who reported less than one minute of physical activity per week (16.6% missing sitting). Therefore, as it was likely that the data were missing at random [44] and age and physical activity were already included as covariates in the regression models (thereby providing model-based adjustment for the missing data on the sitting outcome) participants without complete data were excluded from the analyses.

#### Statistical analysis

Multiple logistic regression was used to investigate factors associated with higher sitting time for all respondents as well as for four subsamples: participants reporting ever having (a) heart disease, (b) cancer (except non-melanoma skin cancer), (c) diabetes (not further defined), and (d) moderate/high risk of psychological distress. All models included age, gender, education, marital status, work status, urban/rural location of residence, income, BMI, number of co-morbidities, limitation of physical function, self-rated health, physical activity, current smoking status, alcohol consumption and level of risk of psychological distress (except where risk of psychological distress was the outcome). Adjusted OR’s and 95% CI’s are reported and the Wald test used to assess statistical significance. Given the multiple comparisons and the high power to detect very small associations we used a significance level of p<0.01, and considered a 50% increase/decrease in odds (i.e., ORs ≥1.5 or ≤0.67) to be of public health importance [45].

## Results

The mean age of participants was 62.6 years and 55% of participants were female (Table 1), with 44.5% of the sample spending greater than 6 hours per day sitting.

**Table 1:** Distribution of demographic, clinical and health behaviour characteristics for total sample, and by gender (N = 266,826).

	Male %	Female %	Total %
**Demographic**			
Age			
45–54 years	25.3	32.4	29.1
55–64 years	31.6	32.7	32.2
65–74 years	23.9	19.9	21.8
75+ years	19.2	15.0	16.9
Education			
up to year 10	26.4	41.1	34.3
HSC/Tafe/Diploma	48.3	37.0	42.3
Degree or Higher	25.3	21.9	23.4
Marital Status			
Single/Widowed/Divorce	19.1	29.8	24.9
Married/Defacto/partn	80.9	70.2	75.1
Work Status			
Not working	49.9	54.0	52.1
Work p/t	12.8	24.3	19.0
Work f/t	37.3	21.8	29.0
Urban/Non-urban			
Non-urban	46.2	44.1	45.1
Urban	53.8	55.9	54.9
Income			
˂$10,000	4.9	6.2	5.6
$10,000–$29,999	23.8	23.6	23.7
$30,000–$69,999	27.5	23.9	25.6
˃$70,000	27.7	19.9	23.5
Missing	16.2	26.4	21.7
**Clinical**			
Heart Disease			
Yes	16.2	8.2	11.9
Cancer			
Yes	17.3	15.0	16.0
Diabetes			
Yes	11.0	7.3	9.0
BMI			
˂18.5 underweight	0.6	1.6	1.2
18.5-˂25—normal	28.8	38.6	34.1
25-˂30—overweight	44.0	30.1	36.5
30+ obese	20.1	21.0	20.6
Missing	6.5	8.7	7.7
Co-morbidities			
Total score—Mean (SD) (range 0–5)	.45(.68)	.40 (.64)	.43 (.66)
Physical functioning—SF10			
No limitation	30.3	29.2	29.7
Minor limitation	16.6	13.2	14.8
Mild limitation	18.0	15.4	16.6
Moderate limitation	14.2	15.7	15.0
Severe limitation	12.3	15.4	14.0
Missing	8.7	11.1	10.0
Self-rated Health			
Poor	2.4	2.0	2.2
Fair	12.9	11.2	12.0
Good	35.6	32.2	33.8
Very Good	35.9	37.8	36.9
Excellent	13.2	16.8	15.1
Psychological Distress			
Low/no risk	70.6	65.5	67.9
Medium risk	13.1	14.8	14.0
High risk	6.1	7.3	6.8
Missing	10.1	12.5	11.4
**Health Behaviours**			
Sitting Time			
0 –<6 hrs/day	52.7	58.1	55.5
6 hrs +/day	47.4	41.9	44.5
PA Mins/Wk			
˂ 1 min	6.3	7.1	6.7
1–149 mins	19.7	18.2	18.9
150–299 mins	18.5	17.7	18.1
300 mins+	55.5	57.0	56.3
Smoking			
Current smoker	7.6	6.9	7.2
Ex-smoker	44.9	29.3	36.6
Never smoked	47.5	63.8	56.2
Alcohol (drinks)			
0/˂1/week	23.6	41.5	33.2
1–7/week	32.3	37.1	34.9
8–14/week	20.4	15.3	17.7
15+/week	23.8	6.1	14.3

Note: HSC = High School Certificate (i.e., completion of 12 years of schooling).

### Factors associated with higher sitting time

Odds of sitting time was significantly associated with: being male (all respondents/heart disease/cancer sub-samples), older age (all respondents/all sub-samples), high levels of education (all respondents/all sub-samples), being unmarried/partnered (all respondents/heart disease/cancer/psychological distress sub-samples), working fulltime (all respondents/all sub-samples), non-urban living (all respondents/all sub-samples), higher income levels (all respondents/all sub-samples), obesity (all respondents), comorbidities (all respondents/psychological distress sub-sample), physical limitations (all respondents/all sub-samples), poor self-rated health (all respondents/all sub-samples), no physical activity (all respondents/all sub-samples), being an ex-smoker (all respondents), and no alcohol consumption (heart disease sub-sample).

The following factors were significantly associated with higher sitting time based on our meaningful effect size (OR≥1.5): *high income ≥$70*,*000* (whole sample OR = 1.70, 95%CI 1.63–1.78; heart disease subsample OR = 1.89, 95%CI 1.66–2.15; cancer subsample OR = 1.53, 95%CI 1.37–1.71; diabetes subsample OR = 1.86, 95%CI 1.61–2.16; psychological distress subsample OR = 1.73, 95%CI 1.58–1.90); *full-time work* (whole sample OR = 1.90, 95%CI 1.84–1.95; heart disease subsample OR = 1.65, 95%CI 1.50–1.81; cancer subsample OR = 1.87, 95%CI 1.74–2.01; diabetes subsample OR = 1.75, 95%CI 1.58–1.94; psychological distress subsample OR = 1.80, 95%CI 1.70–1.91) and *severe physical limitations* (whole sample OR = 1.63, 95%CI 1.57–1.69; heart disease subsample OR = 1.63, 95%CI 1.46–1.82; cancer subsample OR = 1.65, 95%CI 1.51–1.80; diabetes subsample OR = 1.57, 95%CI 1.40–1.76) (see Table 2).

**Table 2:** Adjusted odds ratios (AOR) with 95% confidence intervals for sitting more than six hours per day for all respondents, respondents who have: ever had heart disease; ever had cancer, ever had diabetes (not further defined); and those at moderate or high risk of psychological distress on the Kessler 10.

	All Respondents n = 227,187			Heart Disease Subsample n = 26,599			Cancer Subsample n = 36,381			Diabetes (NFD) Subsample n = 19,550			Psychological Distress Subsample n = 48,334		
Variable(reference category)	AOR	95% CI	p value	AOR	95% CI	p value	AOR	95% CI	p value	AOR	95% CI	p value	AOR	95% CI	p value
**Sex** **(Male)**															
Female	0.95**	(0.93, 0.96)	p<0.001	0.90**	(0.85, 0.96)	p = 0.001	0.90**	(0.86, 0.94)	p<0.001	0.96	(0.90, 1.03)	p = 0.263	0.95	(0.92, 0.99)	p = 0.028
**Age** **(45–54 years)**			**p<.001** ^1^			**p<.001** ^1^			**p<.001** ^1^			**p = .005** ^1^			**p<.001** ^1^
55–64 years	1.05**	(1.02, 1.07)		1.05	(0.95, 1.16)		1.07	(1.00, 1.15)		1.10	(1.00, 1.21)		1.04	(1.00, 1.09)	
65–74 years	1.09**	(1.05, 1.12)		1.08	(0.97, 1.21)		1.05	(0.97, 1.13)		1.04	(0.94, 1.16)		1.05	(0.99, 1.12)	
75+ years	1.28**	(1.24, 1.33)		1.23**	(1.10, 1.38)		1.24**	(1.13, 1.35)		1.19*	(1.05, 1.34)		1.36**	(1.26, 1.46)	
**Education** **(Up to year 10)**			**p<.001** ^1^			**p<.001** ^1^			**p<.001** ^1^			**p<.001** ^1^			**p<.001** ^1^
HSC/TAFE/Diploma	1.02	(1.00, 1.04)		1.05	(0.99, 1.11)		1.04	(0.99, 1.09)		1.05	(0.99, 1.12)		1.02	(0.97, 1.06)	
Degree or Higher	1.36**	(1.33, 1.40)		1.33**	(1.23, 1.43)		1.31**	(1.23, 1.40)		1.38**	(1.25, 1.51)		1.33**	(1.25, 1.40)	
**Marital Status** **(Single/Widowed/Divorced/ Separated)**															
Married/Defacto/Partner	0.86**	(0.84, 0.87)	p<0.001	0.85**	(0.80, 0.90)	p<0.001	0.84**	(0.80, 0.89)	p<0.001	0.89*	(0.83, 0.95)	p = 0.001	0.82**	(0.79, 0.86)	p<0.001
**Work status** **(Not working)**			**p<.001** ^1^			**p<.001** ^1^			**p<.001** ^1^			**p<.001** ^1^			**p<.001** ^1^
Work part time	1.09**	(1.06, 1.11)		1.09	(1.00, 1.18)		1.07	(1. 00,1.14)		1.04	(0.95, 1.15)		1.07	(1.01, 1.13)	
Work full time	**1.90** **	(1.84, 1.95)		**1.65** **	(1.5, 1.81)		**1.87** **	(1.74, 2.01)		**1.75** **	(1.58, 1.94)		**1.80** **	(1.70, 1.91)	
**Urban/Non-urban**															
Urban	0.79**	(0.78, 0.81)	p<0.001	0.85**	(0.81, 0.90)	p<0.001	0.81**	(0.78, 0.85)	p<0.001	0.86**	(0.81, 0.91)	p<0.001	0.84**	(0.81, 0.87)	p<.001
**Income** **(<$10,000)**			**p<.001** ^1^			**p<.001** ^1^			**p<.001** ^1^			**p<.001** ^1^			**p<.001** ^1^
$10,000 - $29,999	1.14**	(1.09, 1.19)		1.23**	(1.11, 1.36)		1.11	(1.01, 1.22)		1.22**	(1.09, 1.36)		1.15**	(1.06, 1.24)	
$30,000 - $69,999	1.15**	(1.10, 1.20)		1.17*	(1.05, 1.30)		1.07	(0.97, 1.18)		1.21*	(1.07, 1.37)		1.10	(1.01, 1.19)	
$70,000+	**1.70** **	(1.63, 1.78)		**1.89** **	(1.66, 2.15)		1.53**	(1.37, 1.71)		**1.86** **	(1.61, 2.16)		**1.73** **	(1.58, 1.9)	
Income missing	1.02	(0.97, 1.06)		1.03	(0.92, 1.15)		1.01	(0.92, 1.12)		1.00	(0.89, 1.13)		1.00	(0.92, 1.09)	
**BMI** **(˂18.5 underweight)**			**p<.001** ^1^			**p = .028** ^1^			**p<.001** ^1^			**p = .006** ^1^			**p<.001** ^1^
18.5-˂25—normal	1.02	(0.93, 1.10)		0.77	(0.61, 0.97)		1.06	(0.87, 1.29)		0.84	(0.57, 1.25)		1.01	(0.86, 1.18)	
25-˂30—overweight	1.09	(1.00, 1.18)		0.81	(0.64, 1.01)		1.12	(0.92, 1.36)		0.84	(0.57, 1.24)		1.11	(0.95, 1.3)	
30+ obese	1.21**	(1.11, 1.31)		0.85	(0.67, 1.07)		1.20	(0.99, 1.46)		0.95	(0.64, 1.41)		1.24	(1.05, 1.45)	
Missing	1.03	(0.94, 1.13)		0.81	(0.63, 1.03)		1.03	(0.83, 1.26)		0.92	(0.62, 1.38)		1.10	(0.93, 1.31)	
**Comorbidities**	1.03**	(1.02, 1.05)	p<0.001	0.99	(0.95, 1.03)	p = 0.589	0.99	(0.96, 1.03)	p = 0.744	1.01	(0.97, 1.05)	p = 0.607	1.06**	(1.04, 1.09)	p<0.001
**SF10** **(No limitation)**			**p<.001** ^1^			**p<.001** ^1^			**p<.001** ^1^			**p<.001** ^1^			**p<.001** ^1^
Minor limitation	1.07**	(1.05, 1.10)		1.07	(0.96, 1.19)		1.06	(0.98, 1.14)		1.03	(0.92, 1.15)		0.99	(0.93, 1.06)	
Mild limitation	1.14**	(1.11, 1.17)		1.18*	(1.07, 1.30)		1.10	(1.02, 1.18)		1.16*	(1.04, 1.29)		1.03	(0.97, 1.10)	
Mod limitation	1.23**	(1.19, 1.27)		1.27**	(1.15, 1.4)		1.25**	(1.16, 1.34)		1.27**	(1.14, 1.41)		1.08	(1.01, 1.15)	
Severe limitation	**1.63** **	(1.57, 1.69)		**1.63** **	(1.46, 1.82)		**1.65** **	(1.51, 1.80)		**1.57** **	(1.40, 1.76)		1.44**	(1.34, 1.54)	
Missing	0.88**	(0.85, 0.92)		0.94	(0.82, 1.07)		0.97	(0.88, 1.07)		0.93	(0.81, 1.07)		0.85**	(0.78, 0.93)	
**Self-rated Health**	0.93**	(0.92, 0.94)	p<0.001	0.90**	(0.87, 0.93)	p<0.001	0.91**	(0.89, 0.94)	p<0.001	0.88**	(0.84, 0.91)	p<0.001	0.91**	(0.89, 0.94)	p<0.001
**PA** **(˂1 min)**			**p<.001** ^1^			**p<.001** ^1^			**p<.001** ^1^			**p<.001** ^1^			**p<.001** ^1^
1–149 mins	0.89**	(0.86, 0.93)		0.77**	(0.69, 0.85)		0.84**	(0.77, 0.93)		0.77**	(0.69, 0.87)		0.84**	(0.78, 0.90)	
150–299 mins	0.90**	(0.87, 0.94)		0.76**	(0.68, 0.85)		0.84*	(0.76, 0.93)		0.76**	(0.68, 0.86)		0.85**	(0.78, 0.92)	
300+ mins	0.73**	(0.70, 0.76)		0.61**	(0.55, 0.67)		0.69**	(0.63, 0.76)		0.62**	(0.55, 0.69)		0.66**	(0.61, 0.71)	
**Smoking** **(Current smoker)**			**p<.001** ^1^			**p = .058** ^1^			**p = .003** ^1^			**p = .012** ^1^			**p<.001** ^1^
Ex-smoker	1.07**	(1.03, 1.11)		0.95	(0.85, 1.08)		0.98	(0.89, 1.08)		0.98	(0.87, 1.10)		1.06	(1.00, 1.13)	
Never smoked	0.99	(0.96, 1.03)		0.90	(0.80,1.02)		0.91	(0.83, 1.00)		0.89	(0.79, 1.01)		0.96	(0.91, 1.03)	
**Alcohol** **(0/˂1/week)**			**p = .178** ^1^			**p = .005** ^1^			**p = .148** ^1^			**p = .150** ^1^			**p = .322** ^1^
1–7/week	1.00	(0.98, 1.02)		0.91*	(0.86, 0.97)		0.96	(0.91, 1.01)		0.93	(0.87, 1.00)		1.01	(0.96, 1.05)	
8–14/week	1.02	(1. 00,1.05)		0.90*	(0.83, 0.97)		0.99	(0.92, 1.05)		1.01	(0.92, 1.12)		1.04	(0.98, 1.10)	
15+/week	0.99	(0.96, 1.02)		0.90*	(0.83, 0.97)		0.93	(0.86, 0.99)		1.00	(0.9, 1.11)		1.05	(0.99, 1.12)	
**Psychological Distress** **(No/low risk)**			**p<.001** ^1^			**p<.001** ^1^			**p<.001** ^1^			**p<.001** ^1^			
Medium risk	1.03	(1.00, 1.06)		1.09	(1.01, 1.17)		1.07	(1.00, 1.14)		1.00	(0.92, 1.09)		n/a	n/a	
High risk	1.05	(1.01, 1.09)		1.05	(0.95, 1.16)		1.09	(0.99, 1.19)		0.99	(0.88, 1.10)		n/a	n/a	
Missing	0.78**	(0.76, 0.81)		0.79**	(0.73, 0.85)		0.78**	(0.73, 0.84)		0.72**	(0.66, 0.80)		n/a	n/a	

Notes to table:

* p<.01

** p<.001

^1^ Overall test of significance (Wald test) for multiple category categorical variables

NFD = no further definition

**bold** denotes those groups that had an OR >1.5.

## Discussion

Our study identified factors associated with sitting time in (i) general, (ii) chronic disease (heart disease/cancer/diabetes), and (iii) psychologically distressed population groups. To our knowledge, this is the first study to concurrently examine factors associated with sitting time in various at-risk population groups, in a large sample of adults aged ≥45 years. The mean sitting time (5.5 hours/day) in our sample was similar to that of a multi-country population-based European study [17].

Consistent with previous literature [46, 47], our study demonstrates that females were less likely than males to sit for greater than 6 hours per day, in the overall, heart disease and cancer samples. Previous studies examining the association between sitting time and age have included younger age groups than those included in the current study. These studies found that younger age is associated with higher sitting time [15, 18]. In the current study, we found that older age was associated with higher sitting, compared to middle age.

Sitting time in our study was positively associated with higher education in all analyses. In a recent systematic review, the type of sedentary behavior engaged in was an important factor [10], with both television viewing and computer use associated with years of education (albeit in opposite directions), yet overall sitting time was not associated with education level. In all analyses, being married was negatively associated with sitting time. This may be attributable to differences in leisure activities such as television viewing, between married and unmarried individuals. Previous research has demonstrated both positive [48] and negative [8, 49, 50] associations between marital status and television viewing.

The negative association between sitting time and urban location found for all samples is supported by Clark and colleagues [51] who reported that ≥2 hours of television viewing per day was associated with living outside of state capital cities, which may be attributed to environmental factors, such as urban residents having more active travel options and amenities within walking distance, which could result in a reduction of sitting [52].

Obesity was positively associated with high sitting time for the overall sample, as has been found previously for television viewing time [19]. However due to the cross-sectional nature of our study, it is unknown whether high sitting time results in a higher BMI or if those with a higher BMI sit more [20, 53, 54]. Longitudinal studies are required to further investigate the direction of the relationship between sitting time and obesity.

Our study appears to be the first to examine the association between chronic disease co-morbidities and sitting time. We found that sitting time was positively associated with chronic disease co-morbidities for the overall and the psychological distress subsample, and negatively associated with self-rated health in all samples, consistent with previous research which demonstrated that television viewing was associated with reduced well-being [10], dissatisfaction with personal life and poorer self-perceived health [7].

In our study more physical activity was associated with lower odds of high sitting time, as previously demonstrated in numerous studies [46, 55, 56]. This highlights the need for interventions that focus on the pattern of physical activity, promoting both increases in physical activity and reductions in prolonged sitting. This might best be achieved by encouraging adults to engage in light intensity activity as well as MVPA, since research shows that adults are more likely to replace sitting behaviors with light intensity activity [57].

High sitting time was positively associated with smoking for the overall sample; a previous review reported mixed findings for the association between smoking and television viewing/general sedentary behavior [10]. In the current study, alcohol consumption was not associated with sitting time which is consistent with most previous findings [19, 58–60]. Interestingly, however, higher alcohol consumption was associated with lower odds of higher sitting time for the heart disease subsample only. It may be that those with heart disease receive more emphasis on physical activity participation and reduced sitting time as a standard part of recovery.

Interestingly, sitting time was not significantly associated with psychological distress, in contrast to previous literature which has identified an association between high levels of sedentary behaviour and greater odds of depression [21, 61]. The K10 assesses psychological distress, rather than depression, which may explain the lack of significance found between psychological distress and sitting time within this study. It may be that factors such as low self-esteem, lack of motivation, feelings of hopelessness and fatigue, which were not examined in our study, may moderate this association [21].

Based on our clinically important effect size of OR≥1.5, *high income* ≥$70,000 (all respondents/all subsamples), *full-time work* (all respondents/all subsamples), and *severe physical limitations* (all respondents/ heart disease/cancer/diabetes subsamples), appear to be associated with highest odds of higher sitting time. Higher sitting time was positively associated with high income categories, which has been previously reported [10]. Evidence suggests that individuals employed in sedentary occupations may also engage in sedentary leisure time activities [62–66].

Higher sitting time was positively associated with full-time work for all subsamples in the current study. Previous literature suggests that individuals who are employed have greater odds of sitting in comparison to those who are unemployed; however sitting time is likely to be influenced by the type of work an individual is involved in [18]. High sedentary time for those in full-time employment is not surprising, considering that the majority of this sedentary time occurs at work [6, 67, 68]. In addition, evidence suggests a positive relationship between unemployment and increased hours of TV also exists [69–72].

Higher sitting time was positively associated with all physical limitation categories (minor to severe), suggesting that odds of sitting time may increase with physical limitation severity; and has been previously demonstrated in cancer [73] and diabetes [16] populations.

We completed a sensitivity analysis using both 7 and 8-hours as cut-points for high sitting time. These results for the 7 and 8-hour cut-points were consistent with all of the 6 hour cut points and OR’s remained at the same level of significance (p<0.001) for each of the study groups for income, hours of full-time work and severe limitations. The OR’s for income within each study group were similar for all three (6, 7 and 8-hour) cut points, while there was a monotonic increase in OR’s for hours of full-time work and severe limitations for the three cut-points.

## Limitations and conclusions

Some study limitations should be acknowledged. First, the cross-sectional nature of the study does not allow the direction of relationships to be determined. While this study may help to identify which chronic disease groups to target, we cannot distinguish which approaches would be most appropriate for each group. Second, self-report measures were used to assess clinical and behavioral variables. Adults tend to under-report body weight [74] and alcohol behavior [75]. Further, the use of a single-item, subjective assessment of sitting time limits the sensitivity/specificity of assessing this behavior, which may have led to under-reporting. To limit response burden in this general health survey, the inclusion of multiple items or objective methods to assess sedentary behavior was not possible. Moreover, light intensity physical activity is difficult to adequately assess with a questionnaire and it may be a modifier of the associations reported in this study. Future studies should employ objective measures where possible for assessing sitting behavior and physical activity and more specific questions including information on domain specific types of sedentary behaviour would also be useful. Third, diabetes type was not differentiated in the questionnaire (e.g., Type 1 or 2), which is important for tailoring interventions.

This study extends the current literature concerning factors associated with high sitting in adults, and appears to be the largest population study to date to examine factors associated with sitting time in both chronic disease (i.e., heart disease, cancer, diabetes) and psychological distress (i.e., anxiety, depression) populations. The identification of key factors from this study may assist to inform the development of future studies using more sophisticated measurement procedures and longitudinal designs to ultimately guide health promotion practice to reduce sitting time**.** It may be particularly important to target high-income, full-time workers and those with severe physical limitations as higher sitting time appears to be substantial in general, chronic disease, and psychologically distressed, adult populations. Future studies are encouraged to examine health effects associated with both duration and type of sedentary behavior, using validated and objective measures, and to undertake longitudinal studies to investigate causal relationships.

## Supporting Information

S1 Data Datasets used to manipulate data and create derived variables and indices. (ZIP)
