# Supplementary material for: Factors Associated with Higher Sitting Time in General, Chronic Disease, and Psychologically-Distressed, Adult Populations: Findings from the 45 & Up Study
Source: PLoS One. 2015 Jun 3;10(6):e0127689. doi: 10.1371/journal.pone.0127689 (PMC4454645; doi:10.1371/journal.pone.0127689)
Supplement: S1 Data — (ZIP) [file pone.0127689.s001.zip › SittingNewcastleVariableCreation&AnalysisForPLoSOneTXT.docx]

* Syntax generated by Anne Grunseit, University of Sydney

*VARIABLE CREATION

label define Sex 1 "Male" 2 "Female"

label values sex Sex

* age cat var

clonevar age_4cat2=agecat

recode age_4cat2 (4=3)

label define age_4cat2 0 "45-54 years" 1 "55-64 years" 2 "65-74 years" 3 "75+years"

label values age_4cat2 age_4cat2

*educat

egen float educat = cut(highestqual), at(0, 3,6,7) icodes

label variable educat "educ categories"

label define educat 0 "up to Year 10" 1 "HSC/TAFE/Diploma" 2 "Degree or higher", replace

label values educat educat

*marital

gen marital=1 if currentmarried==1 | currentpartner==1

recode marital (.=0) if currentsingle==1 | currentwidowed==1 | currentdivorced==1 | currentseparated==1

label variable marital "Marital status"

label define marital 0 "Single/widowed/divorced/separated" 1 "Married/defacto/partner", replace

label values marital marital

*workstatus

gen workstatus=2 if (paidworkhoursnum>=35 & paidworkhoursnum~=.)

recode workstatus (.=0) if paidworkhoursnum==0

recode workstatus (.=1) if (paidworkhoursnum>0 & paidworkhoursnum<=35)

recode workstatus (.=2) if workfulltime==1

recode workstatus (.=1) if workparttime==1

recode workstatus (.=0) if (workfullyretired==1 | workpartretired==1 | ///

workdisabledsick==1 | workselfemployed==1 | workunpaid==1 | workstudyonly==1 ///

| workhomefamily==1 | workunemployed==1 | workother==1)

label define workstatus 0 "not working" 1 "work p/t" 2 "work f/t"

label values workstatus workstatus

*urban_rural

format %8.6f ARIA_plus_mean

gen ASGCRemote= 0 if ARIA_plus_mean <=0.2000001

recode ASGCRemote (.=1) if ARIA_plus_mean>0.2 & ARIA_plus_mean <=2.400

replace ASGCRemote=2 if ARIA_plus_mean>2.40 & ARIA_plus_mean <=5.92

replace ASGCRemote=3 if ARIA_plus_mean>5.92 & ARIA_plus_mean <=10.53

replace ASGCRemote=4 if ARIA_plus_mean>10.53 & ARIA_plus_mean <=15

label define ASGCRemote 0 "Major city" 1 "Inner regional" 2 "Outer regional" 3 "Remote" 4 "Very remote"

label values ASGCRemote ASGCRemote

gen urban_rural=0 if ARIA_plus_mean <=0.2000001

label variable urban_rural "urban_rural"

label define urban_rural 0 "major city" 1 "non-major city", replace

label values urban_rural urban_rural

*income categories

recode income (2=1) (4 3 =2) (5 6 7=3) (8=4) (9 99999 .=5), gen(income5cat_miss)

label define income5cat_miss 1 "<$10k" 2 "$10k-$29999" 3 "$30k-$69999" 4 "$70k+" 5 "inc missing", replace

label values income5cat_miss income5cat_miss

label variable income5cat_miss "inc with missings"

*hadcancer

gen HadCancer2=1 if evertoldbreastyes==1 | evertoldprostateyes==1 | evertoldmelanyes | evertoldothercayes

recode HadCancer2 (.=1) if trtcanceryes==1 & evertoldskinyes==0

recode HadCancer2 (.=0)

label define noyes 0 "No" 1 "Yes"

label variable HadCancer2 "Has/had cancer inc melanoma&other"

label values HadCancer2 cvd noyes

*diabetes

destring evertolddiabetyes evertoldhrtyes

label evertoldhrtyes evertolddiabetyes noyes

* BMI for 45 and up baseline - height and weight limits from HILDA technical report

recode height weight (99999=.)

gen height_mtrs=height/100

gen bmi2_45U= weight/(height_mtrs*height_mtrs)

replace bmi2_45U=. if (sex==1 & height_mtrs <1.3000) | (sex==2 & height_mtrs <1.1000)

replace bmi2_45U=. if (sex==1 & height_mtrs >2.2900 & height_mtrs~=.) | (sex==2 & height_mtrs >2.1000 & height_mtrs~=.) //n=80

replace bmi2_45U=. if (sex==1 & weight <35.0) | (sex==2 & weight <25.0)

replace bmi2_45U=. if (weight >300.0 & weight~=.)

replace bmi2_45U=. if bmi2_45U>50

replace bmi2_45U=. if bmi2_45U<15

egen float bmicat2_45u = cut(bmi2_45U), at(0, 18.5,25,30, 50) icodes

label variable bmi2_45U "bmi 45U no extremes"

label variable bmicat2_45u "bmicats 45U no extremes"

label define bmicat 0 "<18.5 underweight" 1 "18.5-<25-Normal" 2 "25-<30-Overwieght" 3 "30+ obese", replace

label values bmicat2_45u bmicat

clonevar bmicat2_45umiss= bmicat2_45u

recode bmicat2_45umiss (.=4)

label define bmicat 4 "bmi miss", add

label values bmicat2_45umiss bmicat

label variable bmicat2_45umiss "bmi with missings"

*SF10

recode limitvigor-limitdress (99999=.)

*count number of valid values in sf10 vars

egen sf10_num = rownonmiss(limitvigor limitmod limitlift limitupfew limitupone limit1km ///

limithalfkm limit100m limitbend limitdress)

*calculate number of missing values in sf10 vars

gen sf10_nummiss=10-sf10_num

gen limitvigor2 = limitvigor

gen limitmod2 =limitmod

gen limitlift2 =limitlift

gen limitupfew2 =limitupfew

gen limitupone2 =limitupone

gen limit1km2 =limit1km

gen limithalfkm2 =limithalfkm

gen limit100m2 =limit100m

gen limitbend2 =limitbend

gen limitdress2 =limitdress

recode limitvigor2-limitdress2 (3=0) (2=1) (1=2)

label define SF10 0 "not limited" 1 "limited a little" 2 "limited a lot", replace

label values limitvigor2-limitdress2 SF10

egen sf10_tot=rowtotal(limitvigor2 limitmod2 limitlift2 limitupfew2 limitupone2 limit1km2 ///

limithalfkm2 limit100m2 limitbend2 limitdress2) if sf10_nummiss<=5

label variable sf10_tot "SF10 score if <=5missing"

gen SF10_score = 100*((((10*sf10_tot)/(10-sf10_nummiss))-20)/-20)

replace SF10_score=. if sf10_num<5

egen float SF10cat_45U = cut( SF10_score), at(0, 60,85,95,100,101) icodes

recode SF10cat_45U (0=4) (4=0) (1=3) (3=1)

label define SF10cat_45U 0 "no limitation" 1 "minor limitation" 2 "mild limitation" ///

3 "moderate limitation" 4 "severe limitation"

label values SF10cat_45U SF10cat_45U

clonevar SF10cat_45Umiss= SF10cat_45U

recode SF10cat_45Umiss (.=5)

label define SF10cat_45U 5 "SF10 miss", modify

label values SF10cat_45Umiss SF10cat_45U

label variable SF10cat_45Umiss "SF10 cat with missings"

*ratehealth_r

gen ratehealth_r_bi=1 if (ratehealth_R==3 | ratehealth_R==4 | ratehealth_R==5)

recode ratehealth_r_bi (.=0) if (ratehealth_R==1 | ratehealth_R==2)

label define ratehealth_r_bi 0 "poor/fair" 1 "good/vgood/excell"

label values ratehealth_r_bi ratehealth_r_bi

* k10_cat & k10_catmiss

recode feeltired-feelworthless (99999=.)

gen feelsosad2=feelsosad

replace feelsosad2=feeltoodepressed if version_no==1

label variable feelsosad2 "feelsosad+feeltoodepressed"

label values feelsosad2 feelsosad

recode feeltoonervous (.=1) if feelnervous==1

recode feeltoorestless (.=1) if feelrestless==1

recode feelsosad2 (.=1) if feeldepressed==1

egen k10_num = rownonmiss(feeltired feelnervous feeltoonervous feelhopeless feelrestless ///

feeltoorestless feeldepressed feeleffort feelworthless feelsosad2)

gen feeltired2= feeltired

gen feelnervous2= feelnervous

gen feeltoonervous2 =feeltoonervous

gen feelhopeless2 =feelhopeless

gen feelrestless2= feelrestless

gen feeltoorestless2 =feeltoorestless

gen feeldepressed2= feeldepressed

gen feeleffort2= feeleffort

gen feelworthless2=feelworthless

egen k10_mean = rowmean(feeltired feelnervous feeltoonervous feelhopeless feelrestless ///

feeltoorestless feeldepressed feeleffort feelworthless feelsosad2) if k10_num==9

label variable k10_mean "mean K10 if one missing"

replace feeltired2 =k10_mean if k10_num==9 & feeltired==.

replace feelnervous2 =k10_mean if k10_num==9 & feelnervous==.

replace feeltoonervous2 =k10_mean if k10_num==9 & feeltoonervous==.

replace feelhopeless2 =k10_mean if k10_num==9 & feelhopeless==.

replace feelrestless2 =k10_mean if k10_num==9 & feelrestless==.

replace feeltoorestless2 =k10_mean if k10_num==9 & feeltoorestless==.

replace feeldepressed2 =k10_mean if k10_num==9 & feeldepressed==.

replace feeleffort2 =k10_mean if k10_num==9 & feeleffort==.

replace feelworthless2 =k10_mean if k10_num==9 & feelworthless==.

replace feelsosad2 =k10_mean if k10_num==9 & feelsosad2==.

egen k10_score=rowtotal(feeltired2 feelnervous2 feeltoonervous2 feelhopeless2 feelrestless2 ///

feeltoorestless2 feeldepressed2 feeleffort2 feelworthless2 feelsosad2) if k10_num>=9

label variable k10_score "Kessler10 score if <=1missing"

gen k10_cat=1 if k10_score <16

recode k10_cat (.=2) if k10_score >=16 & k10_score<22

recode k10_cat (.=3) if k10_score >=22 & k10_score~=.

label variable k10_cat "Kessler10 score cat"

label define k10_cat 1 "Low/no risk" 2 "Medium risk" 3 "High risk", replace

label values k10_cat k10_cat

clonevar k10_catmiss= k10_cat

recode k10_catmiss (.=4)

label define k10_cat 4 "K10 miss", add

label values k10_catmiss k10_cat

label variable k10_catmiss "K10 cat with missings"

* SitMean_bi

egen sitmean_bi = cut(dayhourssitting),at(0,5.6,25) icodes

label define sitmean_bi 0 "0-5.5hrs/day" 1 "6 hrs+/day", replace

label values sitmean_bi sitmean_bi

*PA4cat840_45U

recode walktimesperweek walktotalhrsweek walktotalminweek vigourtimesperweek vigourtotalhrsweek ///

vigourtotalminweek modtimesperweek modtotalhrsweek modtotalminweek (99999=.)

merge 1:1 studyno_baseline using "U:\ICT_HOMEDRIVE_BACKUP_CF\45 and up\45 & Up Data\padata_45up_ag_211013withkrvars.dta

gen walktimehmin=walktimeh_2*60

gen vigtimeh_dbldmin=vigtimeh_2*60

gen modtimehmin=modtimeh_2*60

recode walktimehmin (840/max=840), generate(walktotalminweek840) copyrest

recode vigtimeh_dbldmin (1680/max=1680), generate(vigdoubled840) copyrest

recode modtimehmin (840/max=840), generate(modtotalminweek840) copyrest

egen PAtotal840_45U=rowtotal(walktotalminweek840 vigdoubled840 modtotalminweek840), missing

egen float PA3cat840_45U = cut(PAtotal840_45U), at(0, 150,299,3361) icodes

label define PA3cat 0 "<150mins" 1 "150-299mins" 2 "300mins+", replace

label values PA3cat840_45U PA3cat

label variable PA3cat840_45U "PA Mins/wk 45U limit 840"

label variable PAtotal840_45U "PA Mins/wk 45U limit 840"

egen float PA4cat840_45U = cut(PAtotal840_45U), at(0, 1, 150,299,3361) icodes

label define PA4cat 0 "<1min" 1 "1-149mins" 2 "150-299mins" 3 "300mins+", replace

label values PA4cat840_45U PA4cat

label variable PA4cat840_45U "PA Mins/wk 45U limit 840"

*smkstatus45U

gen smkstatus45U=1 if (smoeverregyn == 1 | smoagestop~=. | smocigsperday ~=. | smopipecigarperday ~=.)& ///

smoregnowyn == 1 //

recode smkstatus45U (.=2) if (smoeverregyn ==1 | smoagestop~=. | smocigsperday ~=. | smopipecigarperday ~=.) & ///

(smoregnowyn~=1 & smocigsperday ~=0 | smopipecigarperday ~=0) //ex smoker if not missing or 0 & ever but not now

recode smkstatus45U (.=1) if (smoeverregyn ==0 | smoeverregyn ==.) & smoregnowyn ==1 //n=0

recode smkstatus45U (.=2) if smoeverregyn==. & smoregnowyn==. & smoagestop ~=. //n=0

recode smkstatus45U (.=1) if smoeverregyn==. & smoregnowyn==. & ((smocigsperday~=. & smocigsperday~=0) | (smopipecigarperday~=. & smopipecigarperday~=0)) //n=0

recode smkstatus45U (.=3) if smoeverregyn==0 | (smoeverregyn==. & smoregnowyn==0) //n=34481

label define smkstatus 1 "current smoker" 2 "Ex-smoker" 3 "Never smoked", replace

label values smkstatus45U smkstatus

*alcweeklycat

egen float alcweeklycat = cut(alcdrinksperweek), at(0, 1, 8, 15, 141) icodes

label define alcweeklycat 0 "0/<1/week" 1 "1-7/week" 2 "8-14/week" 3 "15+/week", replace

label values alcweeklycat alcweeklycat

label variable alcweeklycat "weekly alc 4 cats"

* DATA ANALYSIS

*Table 1: demographics by sex

foreach var in age_4cat2 educat marital workstatus urban_rural income5cat_miss evertoldhrtyes ///

HadCancer2 evertolddiabetyes bmicat2_45umiss SF10cat_45Umiss ratehealth_r ///

k10_catmiss SitMean_bi PA4cat840_45U smkstatus45U alcweeklycat {

tabulate `var' sex, col nokey nofreq

}

summ comorbid

bysort sex: summ comorbid, det

*Table 2:

*All

logistic SitMean_bi sex i.age_4cat2 i.educat marital i.workstatus urban_rural i.income5cat_miss ///

i.bmicat2_45umiss comorbidities i.SF10cat_45Umiss ratehealth_r ///

i.PA4cat840_45U i.smkstatus45U i.alcweeklycat i.k10_catmiss, or

test 1.age_4cat2 2.age_4cat2 3.age_4cat2

test 1.educat 2.educat

test 1.workstatus 2.workstatus

test 2.income5cat_miss 3.income5cat_miss 4.income5cat_miss 5.income5cat_miss

test 2.smkstatus45U 3.smkstatus45U

test 1.alcweeklycat 2.alcweeklycat 3.alcweeklycat

test 1.bmicat2_45umiss 2.bmicat2_45umiss 3.bmicat2_45umiss 4.bmicat2_45umiss

test 1.PA4cat840_45U 2.PA4cat840_45U 3.PA4cat840_45U

test 1.SF10cat_45Umiss 2.SF10cat_45Umiss 3.SF10cat_45Umiss 4.SF10cat_45Umiss 5.SF10cat_45Umiss

test 2.k10_catmiss 3.k10_catmiss 4.k10_catmiss

*ever had heart disease

logistic SitMean_bi sex i.age_4cat2 i.educat marital i.workstatus urban_rural i.income5cat_miss ///

i.bmicat2_45umiss comorbidities i.SF10cat_45Umiss ratehealth_r ///

i.PA4cat840_45U i.smkstatus45U i.alcweeklycat i.k10_catmiss if evertoldhrtyes ==1, or

test 1.age_4cat2 2.age_4cat2 3.age_4cat2

test 1.educat 2.educat

test 1.workstatus 2.workstatus

test 2.income5cat_miss 3.income5cat_miss 4.income5cat_miss 5.income5cat_miss

test 2.smkstatus45U 3.smkstatus45U

test 1.alcweeklycat 2.alcweeklycat 3.alcweeklycat

test 1.bmicat2_45umiss 2.bmicat2_45umiss 3.bmicat2_45umiss 4.bmicat2_45umiss

test 1.PA4cat840_45U 2.PA4cat840_45U 3.PA4cat840_45U

test 1.SF10cat_45Umiss 2.SF10cat_45Umiss 3.SF10cat_45Umiss 4.SF10cat_45Umiss 5.SF10cat_45Umiss

test 2.k10_catmiss 3.k10_catmiss 4.k10_catmiss

*Had cancer

logistic SitMean_bi sex i.age_4cat2 i.educat marital i.workstatus urban_rural i.income5cat_miss ///

i.bmicat2_45umiss comorbidities i.SF10cat_45Umiss ratehealth_r ///

i.PA4cat840_45U i.smkstatus45U i.alcweeklycat i.k10_catmiss if HadCancer2==1, or

test 1.age_4cat2 2.age_4cat2 3.age_4cat2

test 1.educat 2.educat

test 1.workstatus 2.workstatus

test 2.income5cat_miss 3.income5cat_miss 4.income5cat_miss 5.income5cat_miss

test 2.smkstatus45U 3.smkstatus45U

test 1.alcweeklycat 2.alcweeklycat 3.alcweeklycat

test 1.bmicat2_45umiss 2.bmicat2_45umiss 3.bmicat2_45umiss 4.bmicat2_45umiss

test 1.PA4cat840_45U 2.PA4cat840_45U 3.PA4cat840_45U

test 1.SF10cat_45Umiss 2.SF10cat_45Umiss 3.SF10cat_45Umiss 4.SF10cat_45Umiss 5.SF10cat_45Umiss

test 2.k10_catmiss 3.k10_catmiss 4.k10_catmiss

*ever had diabetes

logistic SitMean_bi sex i.age_4cat2 i.educat marital i.workstatus urban_rural i.income5cat_miss ///

i.bmicat2_45umiss comorbidities i.SF10cat_45Umiss ratehealth_r ///

i.PA4cat840_45U i.smkstatus45U i.alcweeklycat i.k10_catmiss if evertolddiabetyes==1, or

test 1.age_4cat2 2.age_4cat2 3.age_4cat2

test 1.educat 2.educat

test 1.workstatus 2.workstatus

test 2.income5cat_miss 3.income5cat_miss 4.income5cat_miss 5.income5cat_miss

test 2.smkstatus45U 3.smkstatus45U

test 1.alcweeklycat 2.alcweeklycat 3.alcweeklycat

test 1.bmicat2_45umiss 2.bmicat2_45umiss 3.bmicat2_45umiss 4.bmicat2_45umiss

test 1.PA4cat840_45U 2.PA4cat840_45U 3.PA4cat840_45U

test 1.SF10cat_45Umiss 2.SF10cat_45Umiss 3.SF10cat_45Umiss 4.SF10cat_45Umiss 5.SF10cat_45Umiss

test 2.k10_catmiss 3.k10_catmiss 4.k10_catmiss

*anxiety depression

logistic SitMean_bi sex i.age_4cat2 i.educat marital i.workstatus urban_rural i.income5cat_miss ///

i.bmicat2_45umiss comorbidities i.SF10cat_45Umiss ratehealth_r ///

i.PA4cat840_45U i.smkstatus45U i.alcweeklycat if k10_cat==2 | k10_cat==3, or

test 1.age_4cat2 2.age_4cat2 3.age_4cat2

test 1.educat 2.educat

test 1.workstatus 2.workstatus

test 2.income5cat_miss 3.income5cat_miss 4.income5cat_miss 5.income5cat_miss

test 2.smkstatus45U 3.smkstatus45U

test 1.alcweeklycat 2.alcweeklycat 3.alcweeklycat

test 1.bmicat2_45umiss 2.bmicat2_45umiss 3.bmicat2_45umiss 4.bmicat2_45umiss

test 1.PA4cat840_45U 2.PA4cat840_45U 3.PA4cat840_45U

test 1.SF10cat_45Umiss 2.SF10cat_45Umiss 3.SF10cat_45Umiss 4.SF10cat_45Umiss 5.SF10cat_45Umiss

misstable pattern SitMean_bi sex age_4cat2 educat marital urban_rural workstatus income5cat_miss ///

smkstatus45U alcweeklycat bmicat2_45umiss PA4cat840_45U comorbidities ratehealth_r ///

SF10cat_45Umiss k10_catmiss, freq

foreach var in evertolddiabetyes evertoldhrtyes HadCancer2 {

display char(10)

display "Descriptives for `var'"

bysort sex: summ age if `var'==1

tab sex if `var'==1

}

display "Descriptives for k10 mod or high"

bysort sex: summ age if k10_cat==2 | k10_cat==3

tab sex if k10_cat==2 | k10_cat==3

display "Descriptives for all"

summ age

bysort sex: summ age

tab sex

*missings

foreach var in SitMean_bi sex age_4cat2 educat marital workstatus urban_rural income5cat_miss ///

bmicat2_45umiss comorbidities SF10cat_45Umiss ratehealth_r ///

PA4cat840_45U smkstatus45U alcweeklycat k10_catmiss {

tab `var', miss

}

summ dayhourssitting, det

summ dayhourssitting if NewcSitMiss==0, det

tab dayhourssitting

foreach var in sex age_4cat2 SitMean_bi HadCancer2 evertoldhrtyes evertolddiabetyes k10cat_bin2 {

tab NewcSitMiss `var', col chi nokey

}

ttest dayhourssitting, by(NewcSitMiss)

*if missing on sitting

foreach var in SitMean_bi sex age_4cat2 educat marital workstatus urban_rural income5cat_miss ///

bmicat2_45umiss comorbidities SF10cat_45Umiss ratehealth_r PA4cat840_45U smkstatus45U ///

alcweeklycat k10_catmiss HadCancer2 evertoldhrtyes evertolddiabetyes k10cat_bin2 {

tab SitMiss `var' , col chi nokey

}

*for missing on cov or sitting

foreach var in SitMean_bi sex age_4cat2 educat marital workstatus urban_rural income5cat_miss ///

bmicat2_45umiss comorbidities SF10cat_45Umiss ratehealth_r PA4cat840_45U smkstatus45U ///

alcweeklycat k10_catmiss HadCancer2 evertoldhrtyes evertolddiabetyes k10cat_bin2 {

tab NewcSitMiss `var', col chi nokey

}

*for missing on covs

foreach var in SitMean_bi sex age_4cat2 educat marital workstatus urban_rural income5cat_miss ///

bmicat2_45umiss comorbidities SF10cat_45Umiss ratehealth_r PA4cat840_45U smkstatus45U ///

alcweeklycat k10_catmiss HadCancer2 evertoldhrtyes evertolddiabetyes k10cat_bin2 {

tab NewcSitMiss `var' if SitMiss==0, col chi nokey

}

ttest dayhourssitting, by(NewcSitMiss)

ranksum dayhourssitting, by(NewcSitMiss)

median dayhourssitting, by(NewcSitMiss)

*All - with missing

logistic SitMiss sex i.age_4cat2 i.educat marital urban_rural i.workstatus i.income5cat_miss ///

i.smkstatus45U i.alcweeklycat i.bmicat2_45umiss i.PA4cat840_45U comorbidities ratehealth_r ///

i.SF10cat_45Umiss i.k10_catmiss, or

*All - with missing

logistic SitMean_bi sex i.age_4cat2 i.educat marital urban_rural i.workstatus i.income5cat_miss ///

i.smkstatus45U i.alcweeklycat i.bmicat2_45umiss i.PA4cat840_45U comorbidities ratehealth_r ///

i.SF10cat_45Umiss i.k10_catmiss, or

*All - without missing

logistic SitMean_bi sex i.age_4cat2 i.educat marital urban_rural i.workstatus i.income4cat ///

i.smkstatus45U i.alcweeklycat i.bmicat2_45u i.PA4cat840_45U comorbidities ratehealth_r ///

i.SF10cat_45U i.k10_cat, or

*******************************************

*sensitivity analysis with 7 and 8 hour cut-points

foreach var in Sit7hr_bi Sit8hr_bi {

*All

display char(10) "Outcome=`var'"

logistic `var' sex i.age_4cat2 i.educat marital i.workstatus urban_rural i.income5cat_miss ///

i.bmicat2_45umiss comorbidities i.SF10cat_45Umiss ratehealth_r ///

i.PA4cat840_45U i.smkstatus45U i.alcweeklycat i.k10_catmiss, or

*ever had heart disease

logistic `var' sex i.age_4cat2 i.educat marital i.workstatus urban_rural i.income5cat_miss ///

i.bmicat2_45umiss comorbidities i.SF10cat_45Umiss ratehealth_r ///

i.PA4cat840_45U i.smkstatus45U i.alcweeklycat i.k10_catmiss if evertoldhrtyes ==1, or

*Had cancer

logistic `var' sex i.age_4cat2 i.educat marital i.workstatus urban_rural i.income5cat_miss ///

i.bmicat2_45umiss comorbidities i.SF10cat_45Umiss ratehealth_r ///

i.PA4cat840_45U i.smkstatus45U i.alcweeklycat i.k10_catmiss if HadCancer2==1, or

*ever had diabetes

logistic `var' sex i.age_4cat2 i.educat marital i.workstatus urban_rural i.income5cat_miss ///

i.bmicat2_45umiss comorbidities i.SF10cat_45Umiss ratehealth_r ///

i.PA4cat840_45U i.smkstatus45U i.alcweeklycat i.k10_catmiss if evertolddiabetyes==1, or

*anxiety depression

logistic `var' sex i.age_4cat2 i.educat marital i.workstatus urban_rural i.income5cat_miss ///

i.bmicat2_45umiss comorbidities i.SF10cat_45Umiss ratehealth_r ///

i.PA4cat840_45U i.smkstatus45U i.alcweeklycat if k10_cat==2 | k10_cat==3, or

}

tab Sit7hr_bi sex, col nokey

tab Sit8hr_bi sex, col nokey
